# Uncertainty Analysis in Intervention Impact on Health Inequality for Resource Allocation Decisions

**DOI:** 10.1177/0272989X211009883

**Published:** 2021-06-08

**Authors:** Fan Yang, Ana Duarte, Simon Walker, Susan Griffin

**Affiliations:** Centre for Health Economics, University of York, York, Yorkshire, UK; Centre for Health Economics, University of York, York, Yorkshire, UK; Centre for Health Economics, University of York, York, Yorkshire, UK; Centre for Health Economics, University of York, York, Yorkshire, UK

**Keywords:** distributional cost-effectiveness analysis, economic evaluation, health inequality, public health, uncertainty analysis

## Abstract

Cost-effectiveness analysis, routinely used in health care to inform funding decisions, can be extended to consider impact on health inequality. Distributional cost-effectiveness analysis (DCEA) incorporates socioeconomic differences in model parameters to capture how an intervention would affect both overall population health and differences in health between population groups. In DCEA, uncertainty analysis can consider the decision uncertainty around on both impacts (i.e., the probability that an intervention will increase overall health and the probability that it will reduce inequality). Using an illustrative example assessing smoking cessation interventions (2 active interventions and a “no-intervention” arm), we demonstrate how the uncertainty analysis could be conducted in DCEA to inform policy recommendations. We perform value of information (VOI) analysis and analysis of covariance (ANCOVA) to identify what additional evidence would add most value to the level of confidence in the DCEA results. The analyses were conducted for both national and local authority-level decisions to explore whether the conclusions about decision uncertainty based on the national-level estimates could inform local policy. For the comparisons between active interventions and “no intervention,” there was no uncertainty that providing the smoking cessation intervention would increase overall health but increase inequality. However, there was uncertainty in the direction of both impacts when comparing between the 2 active interventions. VOI and ANCOVA show that uncertainty in socioeconomic differences in intervention effectiveness and uptake contributes most to the uncertainty in the DCEA results. This suggests potential value of collecting additional evidence on intervention-related inequalities for this evaluation. We also found different levels of decision uncertainty between settings, implying that different types and levels of additional evidence are required for decisions in different localities.

The value of public health interventions is reflected by their impacts on overall population health and health inequality.^1,2^ Resource allocation decisions in public health could therefore be informed by distributional cost-effectiveness analysis (DCEA),^3^ which extends cost-effectiveness analysis (CEA) to incorporate health inequality concerns. DCEA accounts for between-group differences in the parameters of the evaluation (e.g., the value of inputs in a decision-analytic model) to estimate how an intervention affects health in each population group and then describes its impacts on the overall population health and health inequality.

Uncertainty analysis is an important component of decision analysis. It reflects the uncertainty in the input parameters of the decision model and estimates what this means for the level of confidence in the study results and for decision uncertainty.^4,5^ As with any evaluation, uncertainty in the model parameters of DCEA translates into uncertainty in the overall results and the decision on whether an intervention should be introduced.^6^ The uncertainty in DCEA would imply the decision uncertainty based on an intervention’s impacts on both overall health and health inequality (i.e., the probability of correct conclusions that it will increase overall health and/or reduce inequality).

Evidence on how the value of model inputs varies between population groups of interest (e.g., groups with different socioeconomic status) is a key component in DCEA. Given concerns about degree and quality of such evidence, decision makers would be interested to know whether it is worthwhile obtaining additional evidence to reduce the uncertainty about intervention impacts and thus to support decisions to introduce an intervention. As well as informing whether more evidence is worthwhile in general, it is also important to know on which model inputs further research would be most valuable. Questions about the value of further research could be informed by value of information (VOI) analysis.^6^ Analysis of covariance (ANCOVA)^7^ can be used to explore the correlation between variation in a model input and variation in the estimated intervention impacts on overall health and health inequality.

We have previously adapted a DCEA model of smoking cessation interventions to explore how the assessment of intervention impacts would change when accounting for socioeconomic variations in model inputs.^8^ The model considers the decision in England as a whole and within 2 local government authorities in England. The 2 local authorities, York and Sheffield, represent populations of 205,000 and 573,000 individuals, respectively. They differ in smoking prevalence and population socioeconomic characteristics, with Sheffield having a greater smoking prevalence overall and greater levels of socioeconomic disadvantage compared to York. In England, the responsibility for many aspects of public health policies rests with local authorities, yet many published appraisals of these policies focus only on the national level. Local decision makers may be interested to know whether the conclusions about decision uncertainty based on the national-level estimates could inform local policy and what additional evidence would add most value to reduce the decision uncertainty for their area.

In this study, we show how uncertainty analysis can be employed in DCEA to address the following:

- What is the uncertainty about whether the interventions increase overall health and/or reduce health inequality?
- What is the value of obtaining more information on how model inputs vary with socioeconomic characteristics?
- Among all model inputs incorporating socioeconomic variation, which one contributes most to decision uncertainty?
- How generalizable are conclusions about decision uncertainty and the contribution of different model inputs to decision uncertainty between settings (i.e., England as a whole v. local authority and between local authorities)?

## Methods

An existing DCEA model assessing smoking cessation interventions was extended to incorporate uncertainty around the value of model inputs. To do this, model parameters were characterized as distributions and the uncertainty then propagated through to the model outputs using Monte Carlo simulation (i.e., probabilistic sensitivity analysis [PSA]).

### Model Overview

The model evaluates the costs and health benefits for adult smokers (18–75 years) from the National Health Service (NHS) and personal social services perspective over the individuals’ lifetime.^9^ Socioeconomic status was defined by the Index of Multiple Deprivation (IMD), a weighted composite index combining information from the 7 domains of deprivation (i.e., income, employment, health, education, housing, crime, and living environment) for each small, fixed geographical area of approximately 1,500 residents in England.^10^ Using IMD, we can classify all areas into 5 quintiles, with quintile 1 (IMD1) denoting the most deprived and quintile 5 (IMD5) demoting the least deprived. Each person is allocated to an IMD quintile according to their area of residence. Two active interventions, varenicline^11^ and 7.2 mg e-cigarette,^12^ were compared to “no intervention” and to each other. Varenicline is a prescription medication used to treat nicotine addiction, and e-cigarette is a battery-operated device that delivers nicotine. Both are accessed through primary care. Health benefits are expressed as quality-adjusted life years (QALYs) and costs in pounds sterling (£, 2018 price year) with an annual discount rate of 3.5% applied to both benefits and costs, following the National Institute for Health and Care Excellence (NICE) guidance.^4^

The model is a cohort Markov model, including 3 health states: 1) smokers, 2) former smokers, and 3) death.^13^ The full cohort enters the model via the “smokers” health state and is exposed to the mortality and risk of developing smoking-related diseases. Mortality differs by age and smoking status, with an age-specific relative risk of death by smoking status applied to age-specific all-cause mortality rates. The risk of developing smoking-related diseases also differs by age and smoking status. In each annual cycle, smokers have a probability of quitting smoking (and becoming “former smokers”). Those who receive “no intervention” have a “background” quit rate of 2% (proportion of current smokers who naturally quit each year),^14^ while those who receive the intervention have a higher quit rate, based on the original studies reporting the efficiency of the interventions.^11,12^ Smokers are assumed to receive the intervention in the first year, and intervention costs are applied in the first cycle. From the second cycle, all smokers have the background quit rate, and relapse from former smoker to smoker is not modeled (i.e., the relapse rate is zero). Smokers and former smokers are at risk of 6 smoking-related diseases (modeled as events): lung cancer, coronary heart disease, chronic obstructive pulmonary disease, myocardial infarction, stroke, and asthma exacerbation. Health-related quality of life (HRQoL) in each state is age and smoking status dependent. Each smoking-related disease has associated costs and a disutility (i.e., the decrement in utility due to the impact of the disease). These diseases are modeled as events that occur independently. In each cycle, for the hypothetical cohort, we calculated the number of each disease event that was then multiplied by the event-related costs and associated disutility. The costs were added up to estimate the total costs related to the diseases, and the disutility was combined with health utility to estimate the QALY gained over the cycle.

### Uncertainty in Model Inputs

Socioeconomic variation in model inputs across IMD quintiles was characterized in the model. A brief summary is provided below with details reported elsewhere.^8^ Due to a lack of evidence, uncertainty was assigned to the socioeconomic variation in some model inputs only.

#### Socioeconomic variation in smoking prevalence

The smoking prevalence by IMD quintile, estimated using Public Health England Local Tobacco Control Profiles 2017 data,^15^ was assigned independent β distributions to reflect uncertainty in the PSA (Table 1).

**Table 1:** Parameter Values, Ranges, and Distributions

Characteristic	Mean	95% Confidence Interval	Distribution (Parameter)	Reference
Smoking prevalence			β (α, β)	
IMD1 (most deprived)	17.17%	16.55%, 17.79%	2,441, 11,775	Public Health England Local Tobacco Control Profiles 2017 data^15^
IMD2	15.96%	15.22%, 16.70%	1,516, 7,984	
IMD3	14.09%	13.24%, 14.95%	887, 5,406	
IMD4	12.68%	11.80%, 13.57%	688, 4,733	
IMD5 (least deprived)	11.38%	10.53%, 12.24%	601, 4,676	
Relative risk of death			Lognormal (lm, lv)	
Smokers v. nonsmokers (35–44 years)	1.87	1.34, 2.60	0.63, 0.17	Doll et al.^17^
Smokers v. nonsmokers (45–54 years)	2.28	1.83, 2.83	0.82, 0.11	
Smokers v. nonsmokers (55–64 years)	1.97	1.66, 2.33	0.68, 0.09	
Smokers v. nonsmokers (65–74 years)	1.83	1.57, 2.13	0.61, 0.08	
Smokers v. nonsmokers (75 years)	1.37	1.18, 1.59	0.31, 0.08	
Smokers v. former smokers	1.11	1.04, 1.14	0.09, 0.02	
Relative risk of developing smoking-related diseases			Lognormal (lm, lv)	
IMD1 (most deprived)	1.15	1.06, 1.24	0.137, 0.041	Eberth et al.^18^
IMD2	1.12	1.03, 1.20	0.109, 0.039	
IMD3	1.12	1.04, 1.21	0.114, 0.038	
IMD4	1.08	1.00, 1.17	0.079, 0.039	
IMD5 (least deprived)	1			
Coefficient of HRQoL regression			Multivariate normal	
Age group (16–24 years)	Ref			
Age group (25–34 years)	−0.0124			Health Survey for England data sets (2012 and 2014)
Age group (35–44 years)	−0.0544			
Age group (45–54 years)	−0.0681			
Age group (55–64 years)	−0.0986			
Age group (65–74 years)	−0.107			
Age group (75+ years)	−0.1630			
Former smoker	Ref			
Smoker	−0.0340			
IMD1 (most deprived)	Ref			
IMD2	0.0320			
IMD3	0.0281			
IMD4	0.0545			
IMD5 (least deprived)	0.0736			
Constant	0.903			
Intervention effectiveness			β (α, β)	
Natural quit rate	0.02			
Quit rate of using varenicline	0.19		6, 25	Chengappa et al.^11^
Quit rate of using e-cigarette	0.13		13, 87	Caponnetto et al.^12^
Relative risk of quitting smoking			Lognormal (lm, lv)	
IMD1 (most deprived)	1			Dobbie et al.^20^
IMD2	1.35	0.94, 1.81	0.297, 0.168	
IMD3	1.22	0.79, 1.73	0.195, 0.201	
IMD4	1.27	0.91, 1.67	0.236, 0.154	
IMD5 (least deprived)	1.36	0.94, 1.82	0.308, 0.168	
Service uptake rate			β (α, β)	
IMD1 (most deprived)	4.03%		96, 2,284	Love-Koh et al.^9^
IMD2	6.48%		93, 1,349	
IMD3	6.62%		93, 1,316	
IMD4	10.14%		90, 795	
IMD5 (least deprived)	9.92%		90, 817	

HRQoL, health-related quality of life; IMD, Index of Multiple Deprivation; lm, mean of the log-transformed value; lv, standard deviation of the log-transformed value.

#### Socioeconomic variation in mortality

The annual mortality rates for smokers were estimated using the general population all-cause mortality by age and sex according to IMD quintiles, proportion of smokers, former smokers and nonsmokers, and relative risk of death (see equation (1) for details).

(1) $$\mathrm{Annual}\;\mathrm{mortality}\;\mathrm{rate}\;\mathrm{for}\;\mathrm{smokers}=\frac{\mathrm{All}\;\mathrm{cause}\;\mathrm{annual}\;\mathrm{mortality}\;\mathrm{rate}}{(\mathrm{proportion}\;\mathrm{of}\;\mathrm{smokers}+\frac{\mathrm{proportion}\;\mathrm{of}\;\mathrm{former}\;\mathrm{smokers}}{\mathrm{relative}\;\mathrm{risk}\;\mathrm{of}\;\mathrm{death}\;(\mathrm{smokers}\;\mathrm{vs}\;\mathrm{former}\;\mathrm{smokers})}+\frac{\mathrm{proportion}\;\mathrm{of}\;\mathrm{non}\;\mathrm{smokers}}{\mathrm{relative}\;\mathrm{risk}\;\mathrm{of}\;\mathrm{death}\;(\mathrm{smokers}\;\mathrm{vs}\;\mathrm{non}\;\mathrm{smokers})}}$$

Data on all-cause mortality were extracted from the Office for National Statistics (ONS) data 2010–2015.^16^ Data on proportion of smokers, former smokers, and nonsmokers were estimated previously.^9^ The relative risk of death for smokers v. nonsmokers by age group (35–44, 45–54, 55–64, 65–74, and 75 years) and associated 95% confidence intervals were estimated using mortality data reported in a UK observational study.^17^ The study also provided mortality rates for former smokers to enable estimates of relative risk of death for smokers v. former smokers, although this was not stratified by age, so it was assumed that the relative risk was constant across age groups. Uncertainty in the estimates of all-cause mortality and proportions was not available, and therefore no uncertainty is reflected regarding the underlying mortality rate conditioned on IMD. However, the use of relative risks of death (which are sampled from lognormal probability distributions) allows us to reflect some of the uncertainty in these estimates. Details are presented in Table 1.

#### Socioeconomic variation in smoking-related diseases

Socioeconomic variation in smoking-related diseases was considered in the model by assuming that the average incidence of smoking-related diseases, reported separately for smokers and former smokers,^9^ was representative of incidence in IMD3. By applying relative risks of developing these diseases in other quintiles compared to IMD3,^18^ we estimated incidence by IMD quintile. Although uncertainty was associated with the relative risks (Table 1), a variance-covariance matrix for the regression model was not reported to allow for correlation across these parameters. Therefore, independent lognormal distributions were assigned to the log value of each of the relative risks as a second-best alternative to capturing uncertainty in these inputs.

#### Socioeconomic variation in HRQoL

Results from a linear regression model using EQ-5D-3L data from the Health Survey for England data sets^19^ were used to estimate socioeconomic variation in HRQoL. The variance-covariance matrix was extracted, and the corresponding Cholesky decomposition was used to obtain correlated draws from a multivariate normal distribution for use in the PSA. The regression coefficients were applied in the DCEA to estimate HRQoL values disaggregated by smoking status, age, and IMD quintiles (Table 1).

#### Socioeconomic variation in intervention effectiveness

As there was no information on socioeconomic status in the original studies reporting the 12-month quit rates of the interventions,^11,12^ we assumed these were the average effect that could represent that of IMD3 and then used the relative risks of quitting smoking by IMD^20^ to estimate the socioeconomic variation in intervention effectiveness. These relative risks also applied to the no-intervention arm. We assigned β distributions to the average quit rates of the interventions and independent lognormal distributions to the log value of each of the relative risks to reflect uncertainty for the PSA (see Table 1 for details).

#### Socioeconomic variation in intervention uptake

As there were no data on the uncertainty surrounding the estimates about the uptake rate of NHS Stop Smoking Service by IMD,^9^ we assumed a standard error of 10% of mean value and independent β distributions in the PSA (Table 1).

#### Others

Other model inputs for which we considered uncertainty but not socioeconomic variation are displayed in Supplemental Table S1, including costs of interventions, costs of smoking-related diseases, and disutility due to smoking-related diseases. We assumed that a given smoking-related disease event would incur the same costs regardless of IMD. To reflect the uncertainty, we assumed the standard error was equal to 10% of mean value and assigned gamma distributions. The disutility due to each disease event was applied as absolute decrements to the baseline HRQoL estimates. Mean estimates and standard errors for disutility were extracted from several studies^21–24^ (see Suppl. Table S1 for details), and gamma distributions were assigned to reflect the uncertainty in the PSA.

### Outcomes of Interest

Our outcomes of interest from the DCEA model were the impact on overall health, measured using population incremental net health benefit (iNHB), and the impact on health inequality, measured using the difference between population incremental “equally distributed equivalent” health (EDE) and population iNHB. Our base population size was 42,994,944 (all adults in England) based on ONS midyear population estimates for 2017.^25^

#### Population iNHB

For each IMD quintile, the model estimates the incremental costs and incremental direct health benefits of providing the active interventions compared to no intervention. If an intervention is implemented that requires additional resources, there would be forgone health associated with not using that funding for other health-improving services (i.e., health opportunity costs). To capture the impact of the intervention on each IMD quintile, the distribution of the health opportunity costs also needs to be considered. As a marginal increase in the NHS budget is expected to be spent more on treating deprived groups, the health opportunity costs of increased spending on other activities fall more heavily on the more deprived.^26^ Using the lower bound of the NICE cost-effectiveness threshold of £20,000^27^ and the proportion of the health opportunity costs borne by each IMD,^26^ incremental costs are converted to the health opportunity costs by IMD and then subtracted from the incremental direct health benefits to obtain the iNHB for each IMD quintile. The impact on overall health is the sum of the iNHB across all quintiles. An intervention with a positive population iNHB is considered to improve overall health.

#### Population incremental EDE

The value of the distribution of health across IMD quintiles can be described using 1 measure, EDE health. We derive EDE health using an Atkinson index,^28^ for which we inform the inequality aversion parameter based on the strength of the general public’s preference toward reducing health inequality, elicited from a population survey in England.^29^ Bellù and Liberati^30^ provide a step-by-step tutorial to calculating the Atkinson index and the EDE for the distribution of income, and we apply that process to the distribution of health. EDE health adjusts the value of overall population health according to the level of inequality in its distribution and the level of inequality aversion. We calculate EDE health with “no intervention” using evidence on the baseline distribution of health measured using quality-adjusted life expectancy (QALE).^31^ (QALE is a measure of life expectancy that is weighted by the health-related quality of life; i.e., it represents the number of QALYs an individual is expected to experience over their lifetime from birth.) For the 2 active interventions, we add the iNHB for each IMD quintile to the corresponding baseline QALE in that quintile. This provides the predicted distribution of health following the implementation of the interventions, which can also be summarized by the EDE health. By subtracting the baseline EDE health from the EDE health with the intervention, we calculate the change in population EDE health due to the provision of active intervention (i.e., population incremental EDE [iEDE]). A positive change in EDE could be the result of an increase in overall health, a reduction in health inequality, or both. To isolate the impact on health inequality, the difference between iEDE and iNHB is used. An intervention with a positive difference (iEDE-iNHB >0; i.e., iEDE > iNHB) is considered to have reduced health inequality.

We can also calculate the same metrics for the comparison between the 2 active interventions. Detailed information on the calculation of iNHB and iEDE is available in a separate publication.^8^

### Uncertainty Analysis

PSA was performed using Monte Carlo simulation (1,000 simulations). For each simulation, population-level iNHB and (iEDE-iNHB) were estimated for all the pairwise comparisons. The uncertainty in the results was evaluated using the probability of increasing overall health (iNHB >0) and the probability of reducing health inequality (iEDE > iNHB). Results were presented visually as scatterplots on the “health equity impact plane.”^32^ This plane illustrates impacts on overall health and inequality simultaneously, with the y-axis indicating the impact on overall health (here population iNHB) and the x-axis indicating the impact on health inequality (here the difference between population iEDE and population iNHB). An intervention that improves overall health (iNHB >0) falls in the upper side of the plane. An intervention that reduces inequality (iEDE > iNHB) falls in the right side of the plane.

The contribution of uncertainty in each group of associated model inputs was assessed using 2 methods: VOI analysis via the Sheffield Accelerated Value of Information (SAVI) platform^33^ and analysis of covariance (ANCOVA).^7^ VOI can be conducted including expected value of perfect information (EVPI) and expected value of partial perfect information (EVPPI)^6^ to estimate the monetary value of resolving all of the decision uncertainty related to all parameters (EVPI) or a subset of parameters (EVPPI).^34^ In this study, EVPI and EVPPI for the total population were calculated. ANCOVA captures the relative effect of the variation in model inputs to the variation in the results by fitting a general linear regression model.^7^ It is expected that parameters that explain most variation in model outputs would also be the ones that contribute most to decision uncertainty. The 1,000 sets of input values and corresponding outcomes from the Monte Carlo simulations were recorded for input into SAVI to undertake VOI and to facilitate linear regression for ANCOVA. For VOI, the overall EVPI and EVPPI for each subset of parameters were reported, and for ANCOVA, the proportion of sum of squares explained by variation in input parameters was reported, both with a higher value indicating more importance for determining uncertainty/variation in outputs.

### Local Authority-Level Analysis

**Figure 1:**
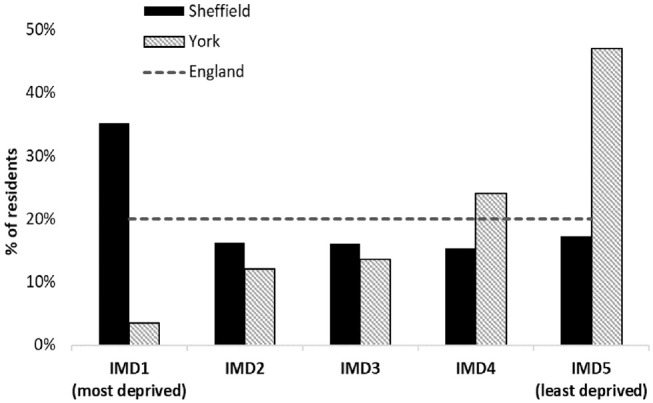
Population distribution according to Index of Multiple Deprivation (IMD) in York and Sheffield.

The analysis was also conducted for 2 local authorities (York and Sheffield) considering the differences in smoking prevalence and associated uncertainty^15^ (Table 2), the different population sizes (York: 207,000 and Sheffield: 574,000), and distributions of socioeconomic groups^35^ (Figure 1). Uncertainty in the other model inputs was based on those for England in the absence of relevant data at the local level.

**Table 2:** Smoking Prevalence at Local Authority

Characteristic	Mean, %	95% Confidence Interval, %	Reference
York			
IMD1 (most deprived)	16.91	11.86, 21.96	Public Health England Local Tobacco Control Profiles 2017 data^15^
IMD2	14.56	9.95, 19.16	
IMD3	13.57	9.19, 17.96	
IMD4	11.64	7.62, 15.66	
IMD5 (least deprived)	10.78	6.95, 14.60	
Sheffield			
IMD1 (most deprived)	22.27	15.86, 28.68	Public Health England Local Tobacco Control Profiles 2017 data^15^
IMD2	20.60	14.47, 26.72	
IMD3	19.84	13.89, 25.79	
IMD4	18.45	12.74, 24.16	
IMD5 (least deprived)	17.74	12.21, 23.28	

IMD, Index of Multiple Deprivation.

## Results

### Base Case

The base case results at the national level are presented in Table 3. Compared to no intervention, both active interventions were estimated to improve overall health (iNHB: 123,749 QALYs [varenicline]; 80,782 QALYs [e-cigarette]) but increase health inequality (iEDE-iNHB: –17,196 QALYs [varenicline]; –10,780 [e-cigarette]).

**Table 3:** Estimates of Intervention Impacts

Region	Intervention	Impact on Overall Health	Impact on Health Inequality	Probability (%) of	
		(iNHB, QALYs)	(iEDE-iNHB, QALYs)	iNHB >0	iEDE > iNHB
England	Varenicline v. no intervention	123,749	−17,196	100.00	0.00
	E-cigarette v. no intervention	80,782	−10,780	100.00	0.00
	Varenicline v. e-cigarette	42,968	−6,417	76.20	19.40
York	Varenicline v. no intervention	659	−9	100.00	38.70
	E-cigarette v. no intervention	431	3	100.00	57.90
	Varenicline v. e-cigarette	229	−11	76.00	20.40
Sheffield	Varenicline v. no intervention	2,092	−467	100.00	0.00
	E-cigarette v. no intervention	1,365	−303	100.00	0.00
	Varenicline v. e-cigarette	727	−164	76.20	22.20

iEDE, incremental equally distributed equivalent health; iNHB, incremental net health benefit; QALY, quality-adjusted life year.

Compared to e-cigarette, varenicline was estimated to increase overall health (iNHB: 42,968 QALYs) but increase health inequality (iEDE-iNHB: –6,417 QALYs).

### Uncertainty Analysis

**Figure 2:**
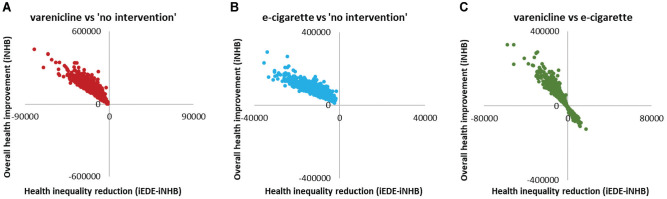
Scatterplots on equity impact plane for all adults in England (*n* = 42,994,944). iEDE, incremental equally distributed equivalent health; iNHB, incremental net health benefit.

PSA results are presented in Table 3 and Figure 2.

**Figure 3:**
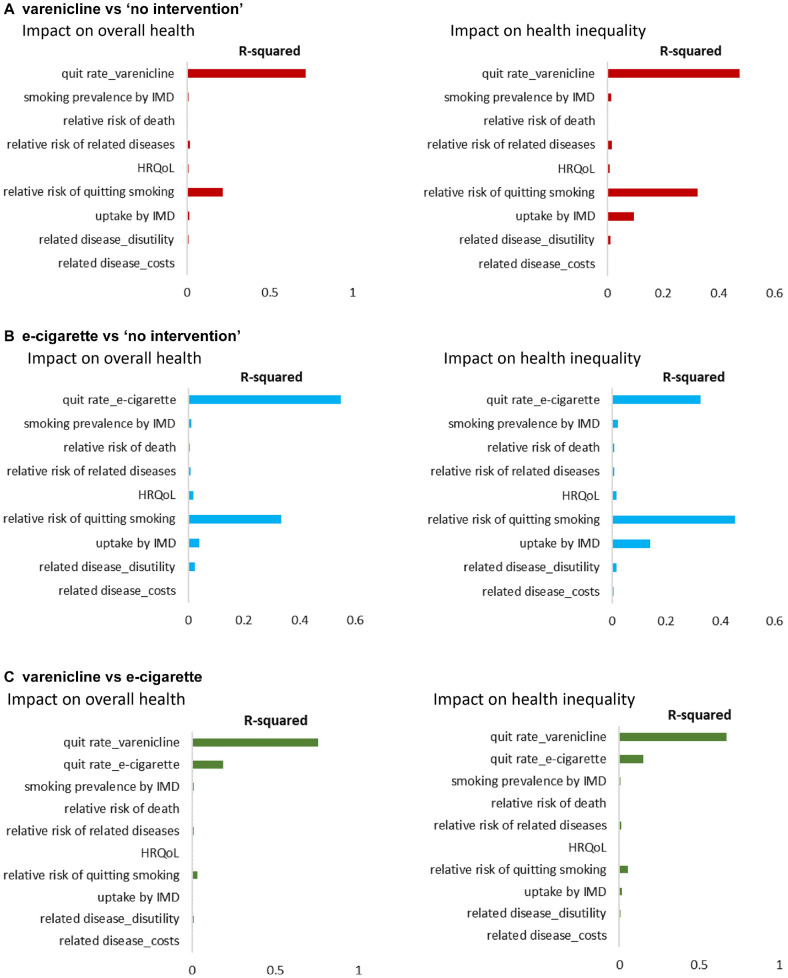
Analysis of covariance (ANCOVA) results. HRQoL, health-related quality of life; IMD, Index of Multiple Deprivation.

For the comparisons between active interventions and no intervention, the probability of improvement in overall health (iNHB >0) was 100%, and the probability of reduction in health inequality (iEDE > iNHB) was 0% (Table 3), suggesting no uncertainty around the conclusion that provision of varenicline or e-cigarette would increase overall health but increase inequality (Figure 2a,b). VOI analysis was not performed as there was no decision uncertainty. ANCOVA results showed that the variation in overall health impact was mainly explained by the variations in the intervention average quit rate and in the relative risks of quitting smoking between socioeconomic groups; the variation in health inequality impact was explained by these and also by the variation in the intervention uptake rates between groups (Figure 3a,b).

**Figure 4:**
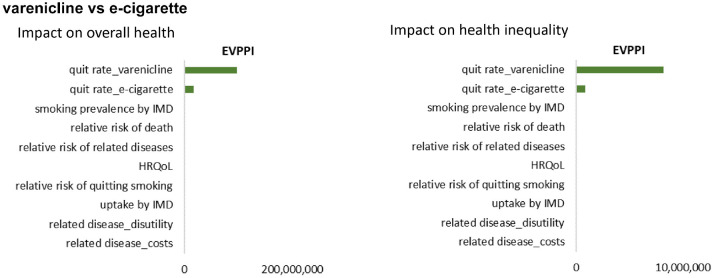
Expected value of partial perfect information (EVPPI) results of comparison between varenicline and e-cigarette. HRQoL, health-related quality of life; IMD, Index of Multiple Deprivation.

For the comparison between varenicline and e-cigarette, there was uncertainty as to whether varenicline increases overall health (probability of 76.20%) and reduces health inequality (probability of 19.40%) compared to e-cigarette (Table 3). The overall population EVPI for the impact on overall health and for the impact on health inequality, estimated by VOI analysis, was £136,312,000 and £12,847,000, respectively. The £136 million demotes the value of eliminating all uncertainty from the analysis about which active intervention improves overall health, and the £12 million denotes the value of eliminating all uncertainty about which active intervention reduces health inequality (Suppl. Table S2). Figure 4 presents the EVPPI results that the uncertainty in both impacts was mainly determined by the uncertainty in the average quit rate of varenicline and in the average quit rate of e-cigarette. None of the uncertainty in socioeconomic pattern of parameters appears to contribute to the uncertainty in the results of the comparison between the 2 active interventions. The results from the ANCOVA (Figure 3c) are similar.

### Local Authority

**Figure 5:**
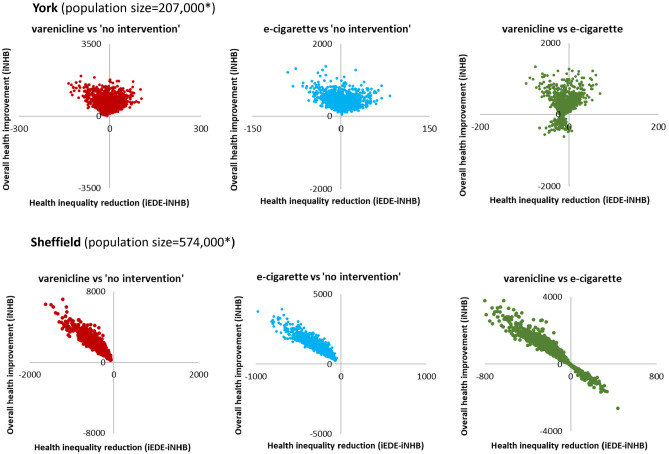
Scatterplots on equity impact plane in York and Sheffield. iEDE, incremental equally distributed equivalent health; iNHB, incremental net health benefit. *The population sizes are rough approximation, rounded to the nearest thousand.

Table 3 also presents the base case and PSA results for York and Sheffield. Compared to no intervention, both active interventions were estimated to increase overall health, and there was no uncertainty around this conclusion. However, for the impact on health inequality, the conclusion about whether the intervention increases/reduces inequality differed, and there were different levels of uncertainty around it between settings (Table 3 and Figure 5). In York, varenicline was estimated to increase inequality with the probability of being inequality reducing at 38.70%, compared to no intervention, while e-cigarette was estimated to reduce health inequality with the probability of being inequality reducing at 57.90%. In Sheffield, both active interventions were estimated to increase overall health and increase inequality with no uncertainty.

For the comparison between varenicline and e-cigarette, there was uncertainty as to whether varenicline increases overall health (76.00% in York and 76.20% in Sheffield) and reduces health inequality (20.40% in York and 22.20% in Sheffield) compared to e-cigarette (Table 3).

ANCOVA and VOI results showed consistent results as that observed for England, with the exception that the uncertainty in smoking prevalence also contributed to the uncertainty about reduction in inequality of active interventions compared to no intervention at the local authority level (Suppl. Figures S1–S4). In York, the uncertainty in smoking prevalence also explained the uncertainty in inequality impact of varenicline compared to e-cigarette (Suppl. Figures S1 and S3). Overall EVPI and EVPPI estimates for both local authorities are available in Supplemental Tables S3 to S5.

## Discussion

In this study, we demonstrate for the first time how uncertainty analysis can be employed when assessing an intervention’s impacts on both overall population health and health inequality. The probability of the intervention being health-improving and the probability of being health inequality-reducing were estimated, to characterize the level of confidence in the qualitative conclusions about the intervention impacts (increase/reduce population health and reduce/increase inequality). The analysis we performed here provides guidance for future studies to apply uncertainty analysis in DCEAs. As socioeconomic variation in model inputs was found to affect the DCEA model outputs,^8^ this study furthers our understanding by exploring how and to what extent the uncertainty in socioeconomic variation in model inputs can translate into uncertainty in the estimated intervention impacts.

We found no uncertainty surrounding the conclusion that provision of smoking cessation interventions, varenicline or e-cigarette, is likely to improve overall health and increase health inequality in England. The variation in the estimated impacts was mainly explained by the variation in the average quit rate of the intervention and the variations in the socioeconomic pattern of intervention-related characteristics, that is, how population groups differ in the probability of quitting smoking and how they differ in the intervention uptake rate. Among all model inputs incorporating socioeconomic variation, the uncertainty in intervention-related variations seems to contribute most to the decision uncertainty. This finding would direct efforts to focus limited resources to collect further evidence on these variations to support decision making. It should be noted that the influence of intervention uptake rates on variation of health inequality impact (measured using iEDE-iNHB) was higher than that on variation of overall health impact (measured using iNHB) (Figure 3). This may be because iEDE incorporates inequality while iNHB does not. Increasing uptake in any group will increase iNHB. Increasing uptake of the more deprived will increase iEDE to a greater extent, resulting in less inequality (positive change in [iEDE-iNHB]), while increasing uptake of the less deprived will increase iEDE to a lesser extent, resulting in more inequality (negative change in [iEDE-iNHB]). This means when intervention uptake rates vary, how the overall health impact would change can be predicted by the direction in which the rates vary (increase/decrease), but how the health inequality impact would change depends on in what direction and in which groups (more deprived/less deprived).

For the comparison between the 2 interventions, which share the same socioeconomic pattern in model inputs, uncertainty exists as to which one would improve overall health and reduce inequality. The value of information analysis suggested that there may be value in research that could eliminate these uncertainties. Currently, EVPI for the inequality impact from a DCEA has not been formally used in decision making. The population EVPI value for further research that eliminated uncertainty as to which active intervention reduced health inequality impact was about £12 million. In our example, this denotes the cost of uncertainty about whether the improvement in health measured in EDE QALYs exceeds the improvement in overall health. A decision maker with a strong inequality focus would potentially be interested in this if they would like to be certain that the welfare improvement (measured using iEDE) was worth at least as much if not more than the health improvement (measured using iNHB). It is the upper bound to the value of research to ensure that an intervention is inequality reducing and to avoid the consequences of inadvertently recommending one that is inequality increasing.

When local authority level evidence was considered, the level of uncertainty differed greatly between national and local levels and between the 2 local authorities for the same comparisons. The uncertainty in smoking prevalence was larger at the local level (Table 2) compared to the national figures (Table 1), so it may have been expected that this would have translated into more uncertainty in inequality impact at the local level. However, in Sheffield, there was no uncertainty that providing smoking cessation services is likely to increase inequality. This discrepancy may be explained by the area-specific characteristics used in the model, smoking prevalence (Table 2) and population deprivation structure (Figure 1). For a given individual smoker, smoking cessation is expected to have less success in helping that smoker to quit as the deprivation of the local area increases (Table 1). However, the services still offer an expected improvement in health to all individuals that use them (Suppl. Table S6). Successful quit attempts translate into cost savings for the NHS. Smoking cessation produces fewer additional successful quit attempts per smoker in deprived areas, and therefore such services are less likely to realize cost savings from individual smokers in more deprived areas (Suppl. Table S6). Previous research indicates that cost savings would benefit more deprived areas to the greatest degree.^26^ Thus, smoking cessation is inequality increasing in the distribution of direct health benefits of quitting smoking and inequality reducing in the health benefits from cost saving per smoker. The balance of health gains from smoking cessation services depends on the proportion of the population in each IMD quintile (which can depart from 20% at local level) and the smoking prevalence among residents in each IMD quintile. When estimating the impact at the local authority level, differences in smoking prevalence and population deprivation structure affect the results. To illustrate it, we explored some scenarios (Suppl. Table S7). At the national level, per 100,000 population, the inequality impact (iEDE-iNHB) with varenicline is –40 QALYs. Changing the smoking prevalence in each IMD quintile to that observed in each local area (scenario a) results in a similar change in iEDE-iNHB using York patterns of smoking (–37 QALYs) but a greater discrepancy using Sheffield smoking prevalence (–70 QALYs). Maintaining national smoking rates in each IMD quintile but using each local area population distribution across IMD quintiles (scenario b) would show minimal impact on inequality using the York population structure (–4 QALYs) and a slightly higher impact on inequality compared to the national level using the Sheffield population structure (–50 QALYs). This indicates that in York, the less disadvantaged population structure explains most of the difference, while in Sheffield, both population structure and smoking prevalence explain the difference, but smoking prevalence to the greater degree. Compared to the national-level estimates, local area characteristics imply that providing smoking cessation services is not expected to increase inequality in York but could increase it by a greater degree in Sheffield. Therefore, the inequality impact of the smoking cessation services differs between York and Sheffield. Altogether this implies that estimates of intervention impact on inequality should not be generalized between settings and that setting-specific uncertainty level in model inputs should be considered in DCEAs to inform local decision makers.

We note the limitations of this study. First, although we performed the DCEA at both national and local levels, only local information on smoking prevalence and population distribution was considered. Thus, most of the remaining model inputs with socioeconomic variation were still based on the national figures. As smoking prevalence was found to considerably affect the uncertainty in results, other local-level model inputs may also affect the decision uncertainty to some extent. Further analysis should seek to incorporate more local-level evidence to explore such effect in more detail. Second, the uncertainty surrounding the distribution of health opportunity costs was absent and thus not included in the analysis. It is an important parameter in the calculation of EDE health, so it may have considerable impact on the uncertainty around the intervention’s impact on inequality. Third, when defining the socioeconomic pattern in model inputs, we modeled uncertainty in some between-group differences as independent due to the unavailability of data. For example, we assigned independent distributions to the smoking prevalence across IMD quintiles. Alternative specifications would be worth considering, including those that reflect correlation and dependency, and the possibility of summarizing the socioeconomic pattern with one single parameter. Last, the conclusion that uncertainty in intervention-related socioeconomic variations would drive the uncertainty in estimated intervention impacts was drawn from this single case study only. The model may omit other important socioeconomic differences. For example, socioeconomic variation in relapse rate was not considered. Evidence from South Korea suggests that relapse may be higher in more deprived groups,^36^ which would make the interventions even less effective in the more deprived groups and lead to less favorable results in terms of the potential for the smoking cessation services to reduce inequalities. In the comparison between the 2 active interventions, we used the same socioeconomic pattern in uptake, but there is some evidence that variation in intervention uptake is associated with the type of intervention.^9^ Future applications of the uncertainty analysis in more DCEAs would add to our results to advance our understanding of what uncertainty drives the uncertain conclusions about the intervention impacts. The method we present here for analyzing and presenting the results would still apply.

## Conclusions

Using a DCEA of smoking cessation interventions, our analysis demonstrates that uncertainty analysis within DCEA is feasible and requires little data beyond the requirements of the main analysis. Furthermore, it provides additional information on the confidence level of the conclusions to support decision making. This study found that uncertainty in intervention-related socioeconomic variation would contribute most to the uncertainty in the DCEA results, suggesting potential value of evidence on intervention-related inequality in assessing public health interventions. Our analysis also demonstrates differences in decision uncertainty between settings, suggesting local decisions would be better informed by local-level evidence.

## Supplemental Material

sj-docx-1-mdm-10.1177_0272989X211009883 – Supplemental material for Uncertainty Analysis in Intervention Impact on Health Inequality for Resource Allocation Decisions Click here for additional data file. Supplemental material, sj-docx-1-mdm-10.1177_0272989X211009883 for Uncertainty Analysis in Intervention Impact on Health Inequality for Resource Allocation Decisions by Fan Yang, Ana Duarte, Simon Walker and Susan Griffin in Medical Decision Making
