# Supplementary material for: Uncertainty Analysis in Intervention Impact on Health Inequality for Resource Allocation Decisions
Source: Med Decis Making. 2021 Jun 8;41(6):653–66. doi: 10.1177/0272989X211009883 (PMC8295967; doi:10.1177/0272989X211009883)
Supplement: sj-docx-1-mdm-10.1177_0272989X211009883 – Supplemental material for Uncertainty Analysis in Intervention Impact on Health Inequality for Resource Allocation Decisions [file sj-docx-1-mdm-10.1177_0272989X211009883.docx]

# **Appendices**

**Table S1**. Costs and disutility of smoking-related diseases

|  | **Mean** | **Standard error** | **Source** |
| --- | --- | --- | --- |
| **Intervention costs (annual)** | |  |  |
| Varenicline | £223 | - | Love-Koh et al. 2018 [9] |
| 7.2mg e-cigarette | £43 | - |  |
| **Annual costs** |  |  |  |
| Stroke | 5570 | 557 | Love-Koh et al. 2018 [9] |
| Lung cancer | 9366 | 937 |  |
| MI | 1024 | 102 |  |
| CHD | 1339 | 134 |  |
| COPD | 552 | 55 |  |
| Asthma exacerbation | 1246 | 125 |  |
| **Disutility** |  |  |  |
| Stroke | -0.4839 | -0.0461 | Tengs and Wallace. 2000 [21] |
| Lung cancer | -0.4233 | -0.1003 |  |
| MI | -0.1878 | -0.0334 |  |
| CHD | -0.2409 | -0.0122 | Stevanovic et al. 2016 [22] |
| COPD | -0.2700 | -0.0416 | Rutten-van et al. 2006 [23] |
| Asthma exacerbation | -0.3567 | -0.0694 | Szende et al. 2004 [24] |
| CHD: coronary heart disease, COPD: chronic obstructive pulmonary disease,  MI: myocardial infarction. | | | |

**Table S2**. Overall EVPI and EVPPI of varenicline vs e-cigarette for England*

|  | **Improvement in overall health**  **(iNHB>0)** | **Reduction in health inequality**  **(iEDE>iNHB)** |
| --- | --- | --- |
| Overall EVPI | £136,312,000 | £12,847,000 |
| quit rate_varenicline | £96,865,000 | £8,222,000 |
| quit rate_e-cigarette | £17,409,000 | £859,000 |
| smoking prevalence by IMD | 0 | 0 |
| relative risk of death | 0 | 0 |
| relative risk of related diseases | £47,000 | 0 |
| HRQoL | 0 | 0 |
| relative risk of quitting smoking | 0 | 0 |
| uptake by IMD | 0 | 0 |
| related disease_disutility | 0 | 0 |
| related disease_costs | 0 | 0 |

*rounded to the nearest £k.

EVPI: expected value of perfect information, EVPPI: expected value of partial perfect information,

HRQoL: health-related quality of life, IMD: index of multiple deprivation

iNHB: incremental net health benefit, iEDE: incremental equally distributed equivalent health

**Figure S1**. Analysis of covariance (ANCOVA) results in **York**

| **varenicline vs 'no intervention'** |  |
| --- | --- |
| Impact on overall health  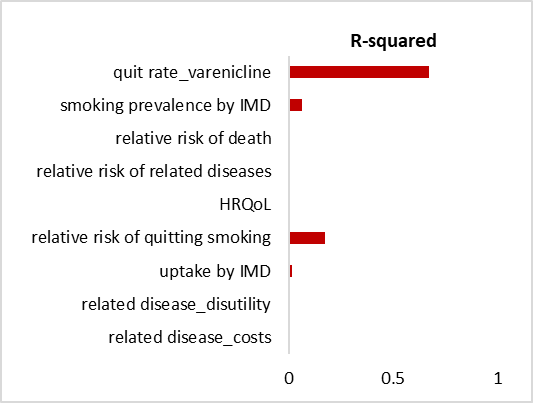 | Impact on health inequality  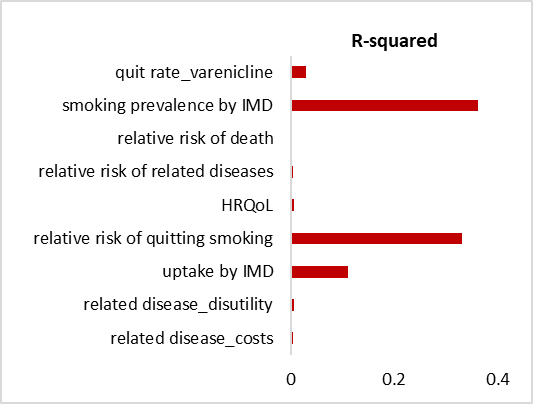 |
|  |  |
| **e-cigarette vs 'no intervention'** |  |
| Impact on overall health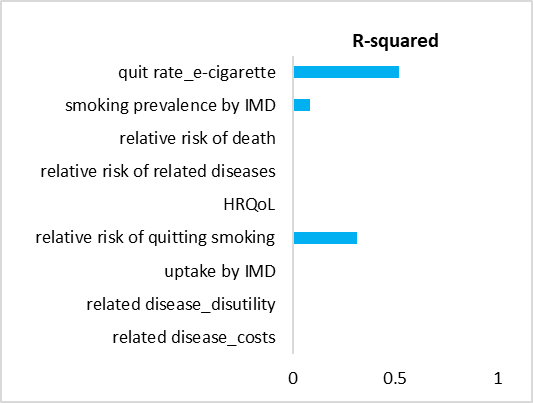 | Impact on health inequality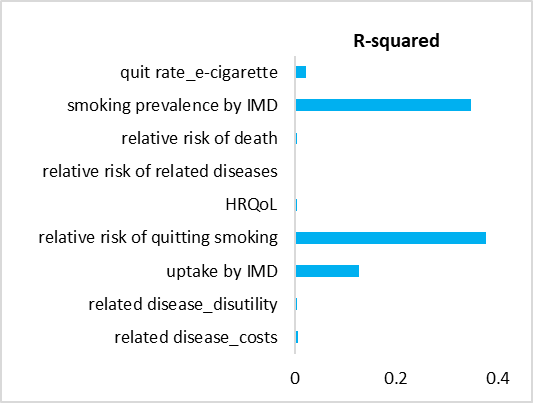 |
|  |  |
| **varenicline vs e-cigarette** |  |
| Impact on overall health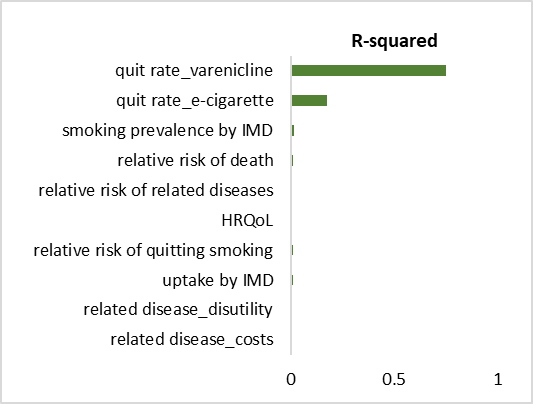 | Impact on health inequality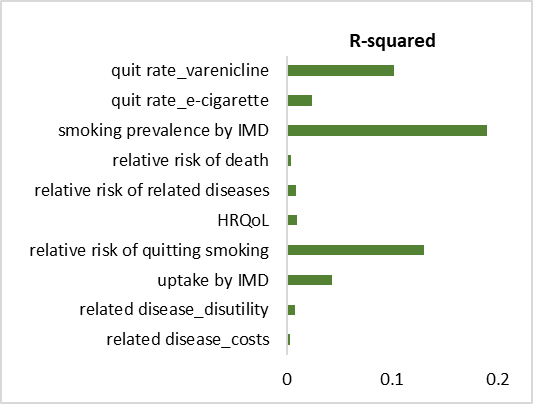 |
|  |  |

**Figure S2**. Analysis of covariance (ANCOVA) results in **Sheffield**

| **varenicline vs 'no intervention'** |  |
| --- | --- |
| Impact on overall health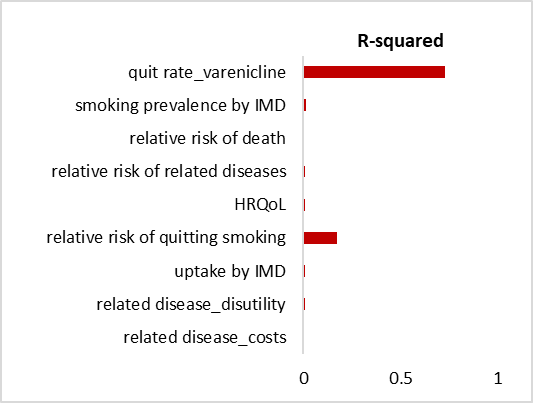 | Impact on health inequality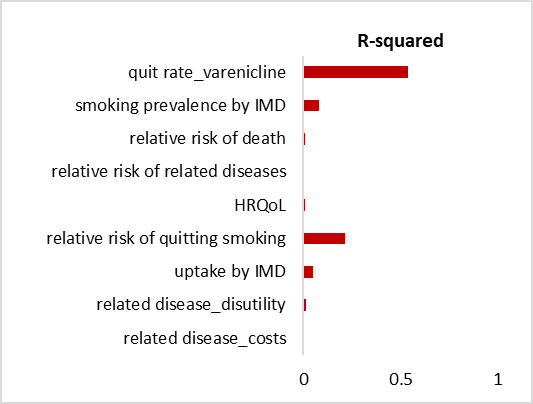 |
|  |  |
| **e-cigarette vs 'no intervention'** |  |
| Impact on overall health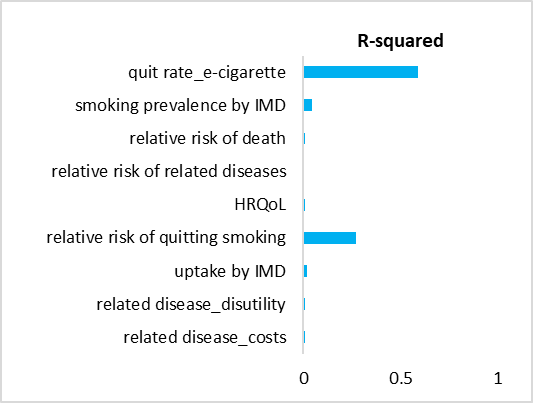 | Impact on health inequality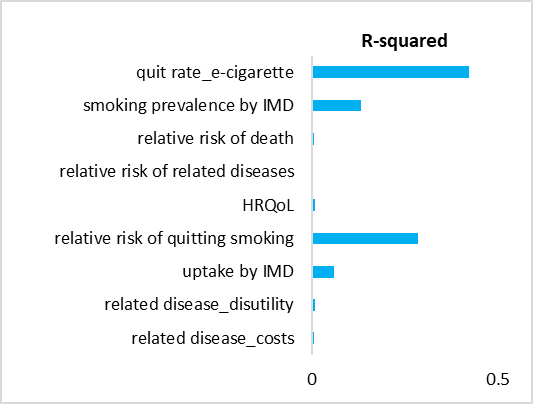 |
|  |  |
| **varenicline vs e-cigarette** |  |
| Impact on overall health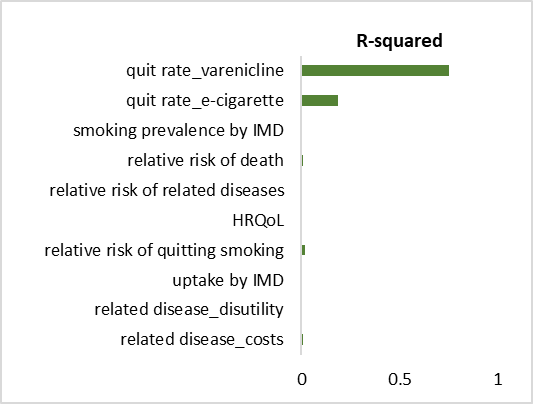 | Impact on health inequality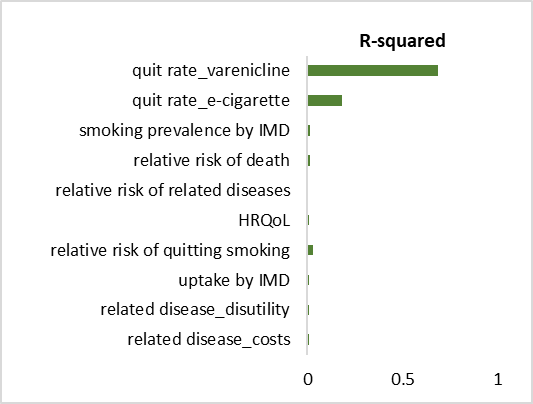 |
|  |  |

**Figure S3**. EVPPI results in **York**

| **varenicline vs 'no intervention'** | **e-cigarette vs 'no intervention'** |
| --- | --- |
| Impact on health inequality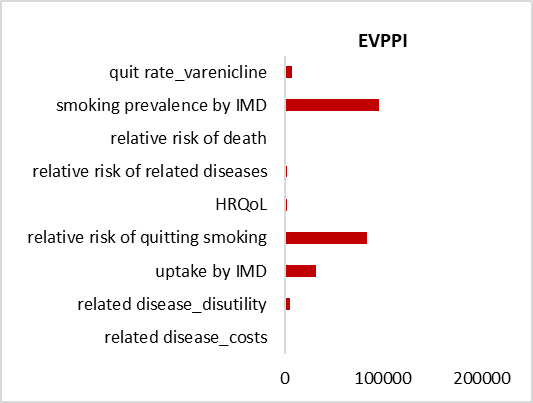 | Impact on health inequality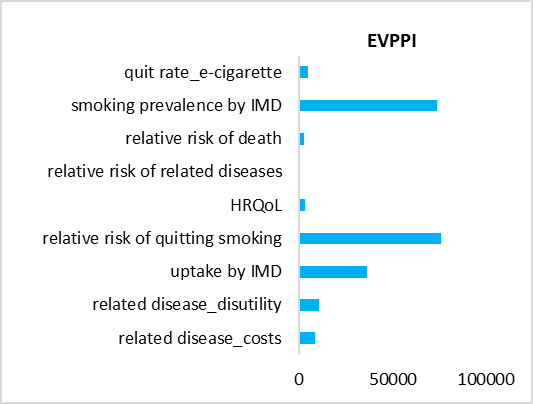 |
| **varenicline vs e-cigarette** |  |
| Impact on overall health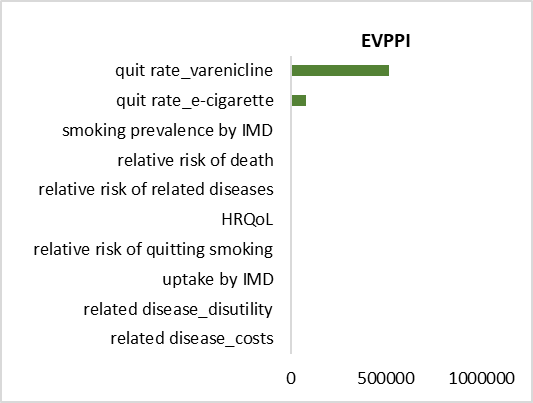 | Impact on health inequality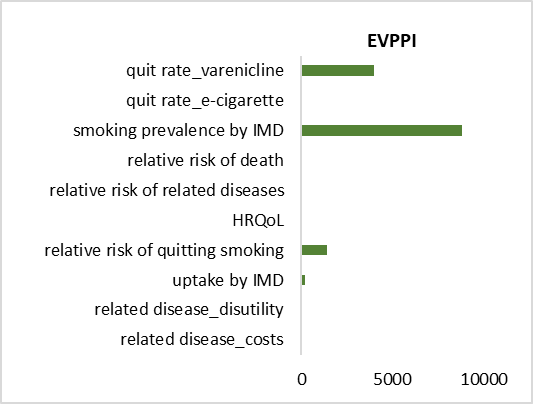 |

**Figure S4**. EVPPI results in **Sheffield**

| **varenicline vs e-cigarette** |  |
| --- | --- |
| Impact on overall health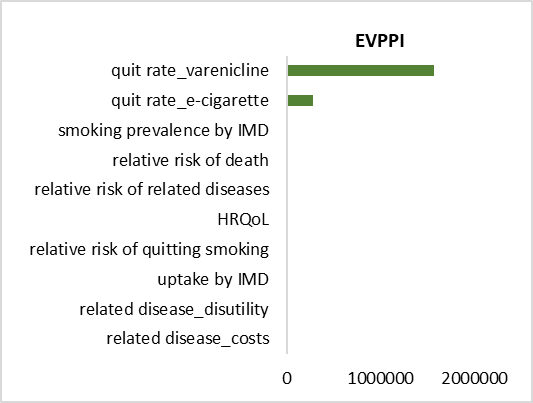 | Impact on health inequality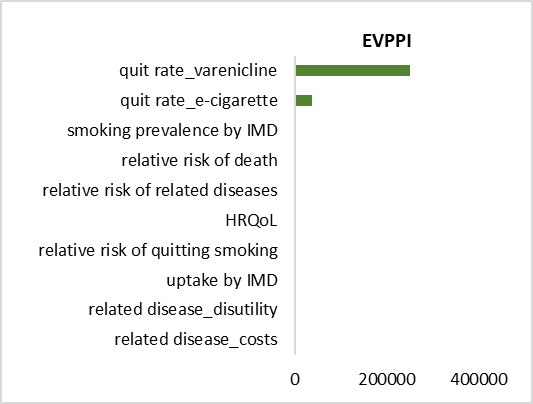 |

**Table S3**. Overall EVPI and EVPPI of active interventions vs ‘no intervention’ for **York***

|  | **Reduction in health inequality (iEDE>iNHB)** | |
| --- | --- | --- |
|  | **varenicline vs 'no intervention'** | **e-cigarette vs 'no intervention'** |
| Overall EVPI | £185,800 | £134,700 |
| quit rate_varenicline/e-cigarette | £7,600 | £4,800 |
| smoking prevalence by IMD | £96,200 | £73,800 |
| relative risk of death | £200 | £2,600 |
| relative risk of related diseases | £2,200 | 0 |
| HRQoL | £2,400 | £3,000 |
| relative risk of quitting smoking | £84,400 | £75,800 |
| uptake by IMD | £32,200 | £36,400 |
| related disease_disutility | £6,000 | £10,600 |
| related disease_costs | £1,200 | £8,600 |

*rounded to the nearest £00.

**Table S4**. Overall EVPI and EVPPI of varenicline vs e-cigarette for **York***

|  | **Improvement in overall health**  **(iNHB>0)** | **Reduction in health inequality**  **(iEDE>iNHB)** |
| --- | --- | --- |
| Overall EVPI | £764,100 | £63,000 |
| quit rate_varenicline | £516,600 | £4,000 |
| quit rate_e-cigarette | £80,800 | 0 |
| smoking prevalence by IMD | 0 | £8,800 |
| relative risk of death | £4,400 | 0 |
| relative risk of related diseases | 0 | 0 |
| HRQoL | 0 | 0 |
| relative risk of quitting smoking | 0 | £1,400 |
| uptake by IMD | £200 | £200 |
| related disease_disutility | 0 | 0 |
| related disease_costs | 0 | 0 |

*rounded to the nearest £00.

**Table S5**. Overall EVPI and EVPPI of varenicline vs e-cigarette for **Sheffield***

|  | **Improvement in overall health**  **(iNHB>0)** | **Reduction in health inequality**  **(iEDE>iNHB)** |
| --- | --- | --- |
| Overall EVPI | £2,369,600 | £415,000 |
| quit rate_varenicline | £1,573,900 | £251,200 |
| quit rate_e-cigarette | £277,200 | £38,000 |
| smoking prevalence by IMD | 0 | 0 |
| relative risk of death | 0 | 0 |
| relative risk of related diseases | 0 | 0 |
| HRQoL | 0 | 0 |
| relative risk of quitting smoking | £2,200 | 0 |
| uptake by IMD | 0 | 0 |
| related disease_disutility | 0 | 0 |
| related disease_costs | 0 | 0 |

*rounded to the nearest £00.

**Table S6**. Incremental costs and incremental health benefits per user of the smoking cessation service

|  | **varenicline vs 'no intervention'** | | **e-cigarette vs 'no intervention'** | |
| --- | --- | --- | --- | --- |
|  | Incremental costs, £ | Incremental  health benefits,  QALYs | Incremental costs, £ | Incremental  health benefits,  QALYs |
| **IMD1 (most deprived)** | -195 | 0.183 | -218 | 0.114 |
| **IMD2** | -401 | 0.267 | -355 | 0.170 |
| **IMD3** | -403 | 0.254 | -354 | 0.161 |
| **IMD4** | -452 | 0.273 | -386 | 0.174 |
| **IMD5 (least deprived)** | -497 | 0.302 | -417 | 0.193 |

**Table S7**. Intervention inequality impact in hypothetical scenarios

|  | **iEDE-iNHB, QALYs per 100,000 residents** | | | | | |
| --- | --- | --- | --- | --- | --- | --- |
|  | **varenicline vs 'no intervention'** | | | **e-cigarette vs 'no intervention'** | | |
|  | England | York | Sheffield | England | York | Sheffield |
| **Base case at national level** | -40 |  |  | -25 |  |  |
| **Scenario a.**  Local smoking prevalence |  | -37 | -70 | -40 | -23 | -44 |
| **Scenario b.**  Area-specific population structure |  | -4 | -50 | -25 | 2 | -32 |
| **Base case at local level** |  | -4 | -81 |  | 1 | -53 |
